# New Method to Motivate Participation in Daily Life/Everyday Life Activities Using Sensor-Based Smart Application Translating Intention into Action (TIA)

**DOI:** 10.3390/s26020539

**Published:** 2026-01-13

**Authors:** Morten Freiesleben, Anina Ritterband-Rosenbaum, Mikkel Damgaard Justiniano

**Affiliations:** Elsass Foundation, Holmegaardsvej 28, 2920 Charlottenlund, Denmark; mo@elsassfonden.dk (M.F.); arr@elsassfonden.dk (A.R.-R.)

**Keywords:** participation, assistive technologies, wearables, inertial measurement unit (IMU), movement sensors, sensor-based, clinical rehabilitation, music, intervention, sense of agency, cerebral palsy (CP)

## Abstract

Background: We explored a new approach for increasing participation in daily life for individuals with severe movement impairments. The core of the approach is an application designed to Translate Intentions into Action (TIA) as a motivational tool for both leisure and clinical training sessions. Methods: The TIA application was developed to enable users to activate motivational feedback, like sounds, music, or videos, through movement measured with an IMU (Inertial Measurement Unit). IMUs were calibrated to user-specific thresholds based on individual movement potential. TIA was tested in two different age groups to assess applicability throughout lifespan and across different motor capacities. Results: The results indicated that TIA can be used for improved participation when positive feedback is provided during the intervention sessions. Observations demonstrated that regardless of age and motor capabilities, increased participation was achieved. TIA demonstrated the far-reaching potential to enhance the engagement and motivation of individuals with different levels of severe disabilities. Conclusions: By providing personalized, positive feedback through movement-activated outputs, TIA can be used by a wide range of people, with or without motor disabilities, to control digital outputs, such as video and audio. These findings suggest that TIA can be a valuable tool in both clinical and leisure settings to promote meaningful participation in activities.

## 1. Introduction

The rising economic burden on the healthcare system is a barrier for providing rehabilitation for people with disabilities throughout lifespan. According to the World Health Organization (WHO) and United Nations Children’s Fund (UNICEF), more than 2.5 billion people worldwide need one or more assistive products [1]. Moreover, the need for assistive devices is projected to reach approximately 3.5 billion people by 2050 due to aging populations and the rising prevalence of noncommunicable diseases [2].

Despite the enormous need, access to assistive devices remains a significant challenge, particularly in low- and middle-income countries. The WHO–UNICEF Global Report on Assistive Technology revealed that nearly one billion people who require assistive products are denied access [1]. This access gap is particularly pronounced in low- and middle-income countries, where as few as 3% of those in need have access to assistive devices, compared to up to 90% in high-income countries [1,2].

Engaging individuals with severe cerebral palsy (CP) who experience multiple impairments and significant restrictions in their ability to participate in everyday activities requires substantial resources in relation to both clinical interventions and leisure activities [3,4]. As a result, individuals with severe CP face extensive challenges associated with participation in typical daily activities on equal terms with their neurologically intact peers. Challenges can be seen across multiple functional variations within the broad spectrum of CP, concerning impairments within cognition, perception, communication, and motor abilities [5]. A better understanding of how to overcome or navigate within or beyond these impairments is essential for participating in activities such as employment, leisure activity, and self-care. There is a well-documented correlation between the level of motor function and the degree of participation, with additional cognitive, communicative, and psychological challenges further limiting opportunities for engagement [6]. For individuals classified within Gross Motor Function Classification Scale (GMFCS) levels III–V, efforts to enhance participation frequently result in mere presence as spectators rather than active involvement [7]. These types of considerations are important for all age groups and functional capacities within the CP population as a common barrier is often the lack of, or a decrease in, meaningful and efficient physical interaction with the environment. A central challenge, which significantly complicates therapy, training, and participation in various activities, is lack of communicational skills and an impaired ability to interact effectively with their surroundings. While specialized training and therapeutic interventions are widely accessible during childhood, adults often encounter a significant lack of support in this area. In particular, interventions aimed at maintaining functional abilities—essential for engaging in everyday activities—are insufficiently provided. This gap contributes to a decline in independent participation and increases reliance on environmental support to engage in meaningful activities. This is where technology can play a crucial role, serving as a catalyst and motivational factor for improved function, activity, and participation, whether in personal performance or social activities [8,9].

Even though there is an access gap globally, a French study showed that 30 to 40% of assistive devices are abandoned during the first year due to lack of adoption, even when assistive technologies are available in high-income countries. This calls for not only a focus on economically accessible solutions, but also solutions with a high level of adaptability and ease of modifications to the user’s specific needs. Furthermore, resources necessary for implementation in individual contexts combined with home-based technologies are also regarded as major barriers [8,10].

Interventions targeting self-initiated goal-directed movements are not a new concept. However, it is essential to allocate sufficient resources to enable practical application and sustainability in home-based settings to support people in improving their functional ability in everyday life activities. This paper introduces Translating Intentions into Action (TIA), a cost-effective and easily accessible wearable technological application designed for home-based training and participation, specifically targeting individuals with motor disabilities such as CP in two different age groups (children and adults). The development of TIA was based on the literature emphasizing the fundamental role of sensorimotor learning, wherein positive and reinforced sensory cues are used for adapting and motivating motor performance and control [11,12]. Furthermore, research underscores the neuroplastic potential of the motor system, which is critically shaped by activity-dependent interactions with the environment from early infancy through childhood and persisting across the lifespan into adulthood [11,13,14,15]. This could provide a window of opportunity to facilitate neurodevelopmental reorganization through sensorimotor activity in play or training sessions for infants and young children with a high risk of a neurodevelopmental disorder by the use of sensor-based wearables. Awareness on how to motivate and engage in increased levels of activity is essential for people with CP of all ages. This calls for allocating rehabilitation resources aimed at reaching milestones for goal-directed, functional training that strengthens sensorimotor development throughout all of life’s activities and not only in clinical settings [8,11].

To demonstrate the applicability and adaptabilities of TIA as a methodological tool for rehabilitation covering the lifespan of people with disabilities, two cases were explored with sensor-based wearable technology. Movement data along the X and Y axes and the TIA application were used to enhance motor behavior in infants and young children with CP, assessed through objective quantification using movement sensor data. Building on this foundation, the application was further developed to support adults with CP in engaging in meaningful activities. This evolution reflects a shift from using TIA solely as an assessment tool to exploring its potential as an assistive device that can actively motivate participation in social and meaningful activities. This is briefly described in the two cases below:

Case 1: We investigated the use of TIA as a rehabilitation tool for infants and young children aged 0–4 years diagnosed with CP or at risk of CP to gain control over their own bodies through cause-and-effect activities, such as activating motivating sounds or images/videos by moving their arms or legs.

Case 2: We focused on adults with severe CP, using TIA as a motivational tool in rehabilitating interventions, by targeting the barriers preventing people with severe motor impairments from participating in meaningful everyday life activities. The aim was to enable participants to play music, either alone or in a group, to increase their level of participation and sense of agency.

The two cases are used to address the following main questions: Can TIA increase the level of participation as a motivational tool via auditory and visual feedback for people with CP in different age groups and with various functional skills? And, furthermore, how do the users perceive TIA in terms of user-friendliness, applicability, and barriers to daily use?

## 2. Materials and Methods

The TIA app facilitates a connection between the user’s input (e.g., movement, pressure, remote sensing) and the desired output (e.g., single sound activation, volume control, music/video playback).

**Figure 1:**
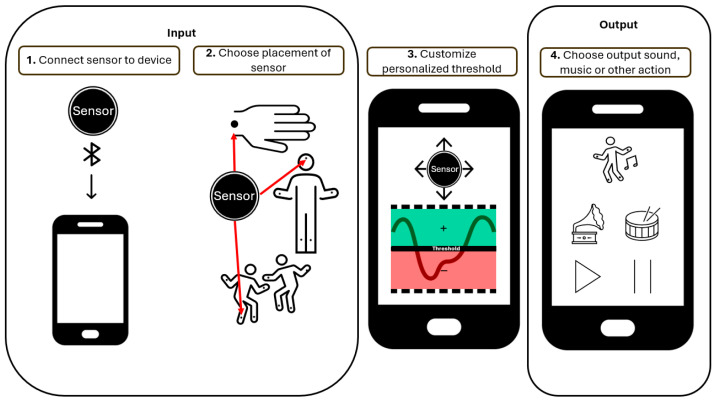
Input–Output model gives an overview of the different parts of the TIA system. A wireless sensor attached to the relevant body part (examples indicated with red arrows in step 2) sends a signal (Input) to the system, which analyses the input based on a given threshold (step 3). Based on a positive (green area) or negative (red area) validation, the sound or action (Output) is controlled (step 4). For a detailed description, see Supplementary Material System Manual S1.

In general, the system can be described as a simple Input–Output model (Figure 1), where the TIA app is the connecting link between the two. The input can be the intension of a user, measured by use of sensors, primarily connecting to the app by means of the Bluetooth Low-Energy protocol, utilizing the Generic ATTribute (GATT) Profile. The advantage over standard Bluetooth is the low energy consumption, the low latency, and the fact that no pairing is needed, making it easy to connect and disconnect devices. The app currently supports various commercial devices, listed in Table 1 together with their available sensors/signals. For ease of use, the app can automatically connect to these nearby known devices.

Each sensor has a fixed set of signals that can be used as input to control the output. Depending on the person’s ability to produce voluntary movements, the sensors can be placed on the preferred extremity or location on the body, e.g., toe, hand, or head. By specifying the type and magnitude of the input, a user-specific setup can be created and adjusted continuously according to the person’s level of motor function. This flexibility enables adjustments of the input threshold required to activate an output. A threshold must be defined beforehand by a short calibration procedure. In addition to the calibrated threshold, an active threshold can be enabled. The threshold will gradually adapt to the user’s level of activity, based on a weighted average of the maximum and average activity during the last five seconds. The mapping from input (sensor signal) to output (sound/action), although no objective quantitative measure is provided, is summarized in the following Algorithm 1:
**Algorithm 1:** Input–Output mappingfunction input2output (*sensorvalue*, *threshold*) **Input:** *sensorvalue*: A sensor value coming from an accelerometer, gyroscope, lightsensor, etc. *threshold*: A threshold value either user-defined or adapted over time **if**
*sensorvalue* > *threshold* **then**   *ActivateOutput*() **else**   *DeactivateOutput*() **end**

For additional information, the source code for the app is available on Github; the link is available in the Data Availability Statement.

With a continuous input signal and the threshold value, the app can be set up to activate several different types of outputs, primarily in the form of auditory and visual stimuli. The app supports the import of sounds and music from the person’s own library. Stimuli are divided into the categories listed in Table 2.

### 2.1. Case 1: Infants and Young Children

A total of 20 children diagnosed with CP or at high risk of CP, aged 0–4 years, participated in 30 min feasibility therapeutic sessions at the Elsass Foundation, Denmark, with the aim of optimizing the technical settings for the TIA app for home-based training tailored specifically to the children’s functional abilities to control the output. Sensors were placed on the children’s extremities to assess movement, and thresholds for movement patterns were adjusted to trigger auditory outputs (sounds that motivated the children, e.g., farting, animals, instruments, etc.). Thresholds and sounds were adjusted throughout the intervention to maximize positive experiences for the children and provide positive and reinforced sensory cues for adapting and motivating increased motor performance.

Observations of the children’s expressions of motivation and levels of self-initiated activity and interviews with parents were conducted during the session. Parents were instructed how to use TIA for home-based training purposes.

### 2.2. Case 2: Adults

In contrast to Case 1, the objective for the adult population was not to evaluate the use of TIA through movement data collection. Instead, the focus was on assessing TIA’s potential as a tool to support independent participation in meaningful activities for adults with CP. Four adults diagnosed with severe CP were recruited. Using the TIA app together with one movement sensor placed on each of the participants extremities, they were able to receive reinforced sensory cues/feedback by moving their arms or legs. The choice of placement was based on either the extremity with the most motor control, or the extremity they wished to involve more actively in motivating activities. The output was presented as a musical instrument in a song of their choice, using the MultiTrack output (Table 2).

Four one-hour group sessions and three individual sessions were conducted. All participants were given time to familiarize themselves with the equipment, determine the appropriate thresholds for sensor activation, and find the optimal placement of the sensors. To ensure high levels of motivation and engagement, close collaboration with the staff and participants was prioritized throughout the sessions.

As described in Case 2, observations were documented to assess expressions of motivation and levels of self-initiated activity. Interviews with the staff were conducted after each session to assess feedback on user friendliness and applicability of TIA.

## 3. Results

This section will elaborate on the potentials of sensor-based wearables in clinical rehabilitation and leisure activities using the TIA application.

### 3.1. Case 1: Infants and Young Children

Results from Case 1 indicated that all children and their parents saw potential for increased motor activity. Personalized setups were created based on each child’s individual needs, including sensor placement, movement thresholds, and motivational outputs, e.g., sounds. All children found the activity motivating, and for the majority of the children, an increased level of self-initiated activity was observed during the 30 min session. In contrast to Case 1, Z axes were recorded and controlled using the TIA system. During the testing phase, we included additional control trials in which the children did not directly engage with TIA. These trials allowed us to compare activity patterns with and without direct interaction with the system. The results showed increased movement across all three axes when the children interacted with TIA and received real-time feedback on their movements, compared to when they were not actively engaging with the system. The increased movement activity was calculated as a ratio and normalized by this formula:

$$\%=\frac{{sensor}_{activity}}{{sensor}_{baseline}}\cdot 100$$

Median value of increased activity = 51% [−21%; 166%]. We found that 16 out of the 18 children had increased activity (positive percentage).

Follow-up interviews indicated that parents found TIA to be a useful motivational tool for increasing self-initiated activity levels. However, while all participating children showed increased engagement and motivation, the successful integration of TIA into daily routines varied among families. Successful implementation required a clear plan detailing how, when, and in which activities to include TIA in their training routines. Some parents reported barriers for integrating new technology into daily activities, primarily due to time and resource constraints in already busy schedules, especially for families with more than one child.

### 3.2. Case 2: Adults

Findings from Case 2 indicated that all four participants perceived TIA as a promising tool for supporting independent participation in playing music. Despite cognitive challenges and variations in sensory perception, each participant successfully activated their instrument, either independently or with verbal guidance from staff.

Although no objective quantitative data were collected from this group, therapists familiar with the participants’ typical behavior and arousal levels provided observational insights. During the sessions, they noted signs of increased engagement, concentration, arousal, and, in some cases, heavy sweating. Positive physical and verbal reactions were given by the participants when they realized that they had the ability to activate their instrument with their own movement repertoire during the song. Evaluation from the participating staff underlined TIA’s ability to create a sense of participation despite a physical disability that prevents the participants from using an ordinary musical instrument. The setup was described as incredibly adaptable, and therefore it was easy to generate a reaction to even the smallest movement, which meant that everyone could participate, despite their motor impairments. It was essential that the imported music was familiar and preferred by the participants, as this considerably enhanced user experience, engagement, and motivation. The different features of the music tracks played a crucial role in providing clear feedback, with heavy beats, distinct melodies, and identifiable instruments. This helped participants distinguish between different sounds and understand the connection between their voluntary movements and the auditory feedback. Genres such as heavy metal, electro, and disco were particularly effective.

## 4. Discussion

The integration of low-cost and adaptive assistive devices into healthcare systems represents a critical frontier in addressing the unmet needs of individuals with disabilities. Despite the potential of assistive technologies to enhance independence, improve quality of life, and reduce healthcare burdens, significant barriers persist in their widespread adoption and impact. The integration of technology into healthcare presents a promising avenue for achieving substantial economic benefits while simultaneously improving patient care and access. Telemedicine can improve access to care, particularly in rural areas, and reduce the financial strain on healthcare facilities [1,17]. It also enables greater independence and increases autonomy for people with disabilities by integrating rehabilitation technologies into the interventions. The emergence of inexpensive IMUs and freely available applications presents a promising opportunity for expanding access to assistive technologies. These innovations not only address the financial barriers that often render advanced rehabilitative tools inaccessible, but also offer the potential to reach a broader population of individuals with disabilities with unmet needs who could greatly benefit from the effects of these technologies [8].

Releasing the solutions as open source and making assistive devices freely available holds the potential to benefit both initial target groups locally, but additionally creates a potential impact for people that do not have the same access to assistive devices. This emphasizes a substantial potential for growth and collective global development of healthcare technologies as free and open solutions. By licensing the TIA application as open source under the MIT license (https://opensource.org/license/mit (accessed on 7 January 2026)), the authors aim to encourage further development and implementation of TIA for a broader population globally.

The TIA application demonstrates potential for use in clinical rehabilitation within the modern health framework outlined by the International Classification of Functioning, Disability, and Health (ICF). The application encompasses three important domains for a comprehensive approach to intervention within the ICF: the functioning of the individual’s developmental body, the activities they are capable of performing, and their participation and engagement in various aspects of life [18]. Results from Case 1 regarding the potential for sensor-based motivational intervention for infants and young children found that TIA can be implemented as an add-on training for children already at a very early age (approximately three months of age) who are at risk of a developmental motor and cognitive disorder such as CP, by potentially enhancing developmental body functions. A few of the small children did not show any increase in activity, which we speculate could be due to the fact that the infants did not understand their role as an agent for controlling the environment. However, as we do not have video recordings for the training sessions, we cannot determine the direct cause for this behavior. TIA promotes increased activity by fostering a heightened sense of agency and understanding of cause and effect and facilitates greater levels of participation for adults by enabling engagement in group music-playing activities. A limitation of the results from the adult population is the lack of quantitative data to support increased activity and participation levels. Therapists working closely with the adults reported the increased level of participation verbally. We recognize that objective quantitative measurements to validate the effect of the use of TIA could have supported the reports of the clinical staff.

Achieving autonomy, independence, and control over one’s own life is linked to a greater quality of life for people with disabilities [19,20]. To enhance the independence of individuals with motor disabilities in various activities, the future integration of TIA applications into smart home technologies holds substantial potential. By enabling users to employ IMUs to control lighting, doors, household appliances, and other devices, TIA facilitates a crucial step towards greater autonomy in daily life activities and during clinical rehabilitation where home-based wearables can monitor physical activity and thereby support potential interventions provided by the therapists [21].

TIA demonstrates great potential across a wide range of motor functions and age groups, indicating its utility in habilitation and rehabilitation interventions throughout the lifespan of individuals with CP. This includes both early intervention for newly diagnosed families and home training across the lifespan. Though the primary target group during the development of the TIA application was individuals with CP, the system’s design and adaptability are intended to accommodate multiple other diagnoses within the spectrum of motor disabilities. Furthermore, the TIA application was developed as a tool for not only clinical purposes but also for engaging people in everyday activities as well. Observations from our adult population made it evident that if the adults were engaged in social activities, the beneficial side effect was increased motor activity reflected in their overall behavior.

It is essential to highlight that implementation of sensor-based devices or new technologies can be challenging due to both societal and individual barriers for adoption [22]. This covers the financial aspects of a particular technology (affordability), the access to technology (accessibility), and the understanding of how to use it and integrate the technology into daily settings (acceptability). Support and close collaboration with end users is required in order to implement the most suitable version of the technology for increased motor activity [22].

According to umbrella reviews evaluating the level of physical activity and the accuracy of consumer wearable technologies in health measurement, it is difficult to evaluate the benefit of increased physical activity, as many studies are inconclusive regarding effectiveness and accuracy of wearable devices in daily settings, often due to heterogeneity regarding diagnosis and specific needs within the target groups [23]. The development of the TIA application supports a wide variety of motor capacities due to the adaptability of the equipment to individual needs. Short-term effects of wearable devices have been evaluated for the two cases, with positive results for increasing and motivating motor performance, but TIA has not been validated for long-term use. Further evaluation of the effects of TIA could be relevant for more personal specific usage based on prolonged monitoring, as seen in the work by Novosel and colleagues [21]. Such solutions would give more integrated information on the person’s behavior and health conditions, similar to functions found in wearables, such as smartwatches. Intelligent interaction between commercial sensor-based devices and new technologies for monitorization and increasing physical activities could change the way health solutions collect and utilize data for facilitating activities in daily life.

## 5. Conclusions

In conclusion, this publication aimed to address whether the TIA application can increase the level of participation as a motivational tool for individuals with CP across different age groups and various functional levels. The two cases of clinical applications for TIA indicate that the TIA application is a promising tool for both facilitating activity for people with motor impairments and giving opportunities for participation in social activities, such as musical ensembles. The overall strength of TIA is its high level of adaptability to each user’s individual motor repertoire and its potential to increase the level of participation across all age groups, from infancy to adulthood, emphasizing TIA’s potential in clinical rehabilitation throughout the lifespan. Furthermore, user feedback highlighted the app’s user-friendliness and applicability, and identified barriers to daily use, such as time constraints in life with disability and the need for a structured plan for implementation. These findings suggest that with adequate support and planning, TIA can be an effective and low-cost addition to home-based rehabilitation programs, providing valuable insights for future improvements and implementations.

## Figures and Tables

**Table 1:** List of supported sensors.

Sensor	Type of Signal(s) Supported	Links and Additional Requirements	Price (€)
Built-in sensor on smart device	Acc, Gyr	None	0 €
Movesense Motion sensors (Movesense Ltd., Vantaa, Finland)	Acc, Gyr, Mag	https://www.movesense.com/product/movesense-sensor-hr2 (accessed on 7 January 2026) Requires firmware update (Gatt_Sensordata) https://bitbucket.org/movesense/movesense-device-lib/src/master/samples/bin/release/ (accessed on 7 January 2026)	99 €
Mbientlab Metamotion (MBIENTLAB INC, San Jose, CA 95124, USA)	Acc, Gyr, Mag, (Bar, Light)	https://mbientlab.com/store/metamotionrl/ (accessed on 7 January 2026)	85 €
Cosmo Switch (FILISIA INTERFACES Ltd., London, UK)	Force	https://www.explorecosmo.com/product/ (accessed on 5 November 2025)	160 €
Retisense Insight (Retisense Inc., Houston, TX, USA)	Force	https://www.retisense.com/product/stridalyzer-insight-sensor-insoles/ (accessed on 7 January 2026)	255 €
Gaitup Physilog 5 (Physilog, 1006 Lausanne, Switzerland)	Acc, Gyr		Quote required
M40 Muscle Sense (Applied Neural Research Corp., Burlington, MA, USA)	EMG	https://www.anrcorp.com/product/m40-muscle-sense/ (accessed on 7 January 2026)	117 €
Puck.JS (Pur3 Ltd., Oxfordshire, UK)	Acc, Gyr	https://shop.espruino.com/puckjslite (accessed on 7 January 2026) Requires custom software flashed to Puck.js (see Supplementary Material Puck.JS code S1.)	32 €

Table of the different commercial sensors supported by the app. Abbreviations: acceleration (Acc), gyroscope (Gyr), magnetometer (Mag), barometer (Bar). For information on links and additional requirements, links to the websites have been included for further information about the product specifications, as well as additional information for specific firmware.

**Table 2:** List of different outputs which can be controlled by the app.

Category	Description of Output
Sound	Plays short sounds/effects, such as animal sounds or percussions on every activation instance.
Music	Plays longer musical samples, where an active signal will start/continue playing, while inactivity will set the sound on hold/pause.
Volume	Increases/decreases the volume of the smart device with respect to activation/deactivation.
Pause/Play	Simulates play/pause control buttons, similar to those on a standard multimedia headset. This can, e.g., be used to control external apps responding to these standard control signals, such as streaming, video, or music apps.
MultiTrack	Increases/decreases the volume of one or more musical files playing in synchrony, based on activation/deactivation. The multiple files can, e.g., be instrumental tracks extracted from a musical sample, using tools such as Spleeter [16] or similar.

## Data Availability

All technical details related to the TIA app can be found on Github: https://github.com/WelfareTech-EF/TIA (accessed on 7 January 2026).

## Supplementary Materials

The following supporting information can be downloaded at https://www.mdpi.com/article/10.3390/s26020539/s1. System Manual S1: “User guide for Translating Intension into Action (TIA)”. Puck.JS code S1: “Movement.js”.

## References

[1] WHO, UNICEF (2022). Global Report on Assistive Technology.

[2] Stephenson J. (2022). Nearly 1 Billion People Globally Who Need Assistive Technologies Lack Access. JAMA Health Forum.

[3] Hirsh A.T., Gallegos J.C., Gertz K.J., Engel J.M., Jensen M.P. (2010). Symptom burden in individuals with cerebral palsy. J. Rehabil. Res. Dev..

[4] Michelsen S.I., Uldall P., Kejs A.M., Madsen M. (2005). Education and employment prospects in cerebral palsy. Dev. Med. Child Neurol..

[5] Engel-Yeger B., Jarus T., Anaby D., Law M. (2009). Differences in patterns of participation between youths with cerebral palsy and typically developing peers. Am. J. Occup. Ther..

[6] Vidart d’Egurbide Bagazgoitia N., Ehlinger V., Duffaut C., Fauconnier J., Schmidt-Schuchert S., Thyen U., Himmelmann K., Marcelli M., Arnaud C. (2021). Quality of Life in Young Adults with Cerebral Palsy: A Longitudinal Analysis of the SPARCLE Study. Front. Neurol..

[7] Imms C., Adair B., Keen D., Ullenhag A., Rosenbaum P., Granlund M. (2016). ‘Participation’: A systematic review of language, definitions, and constructs used in intervention research with children with disabilities. Dev. Med. Child Neurol..

[8] Forman C.R., Nielsen J.B., Lorentzen J. (2021). Neuroplasticity at Home: Improving Home-Based Motor Learning Through Technological Solutions. A Review. Front. Rehabil. Sci..

[9] WHO (2016). Priority Assistive Products List—Improving Access to Assistive Technology for Everyone, Everywhere.

[10] Mensah-Gourmel J., Thepot M., Gorter J.W., Bourgain M., Kandalaft C., Chatelin A., Letellier G., Brochard S., Pons C. (2023). Assistive Products and Technology to Facilitate Activities and Participation for Children with Disabilities. Int. J. Environ. Res. Public Health.

[11] Nielsen J.B., Willerslev-Olsen M., Christiansen L., Lundbye-Jensen J., Lorentzen J. (2015). Science-based neurorehabilitation: Recommendations for neurorehabilitation from basic science. J. Mot. Behav..

[12] Gallagher I.I. (2000). Philosophical conceptions of the self: Implications for cognitive science. Trends Cogn. Sci..

[13] Martin J.H., Friel K.M., Salimi I., Chakrabarty S. (2007). Activity- and use-dependent plasticity of the developing corticospinal system. Neurosci. Biobehav. Rev..

[14] Ritterband-Rosenbaum A., Herskind A., Li X., Willerslev-Olsen M., Olsen M.D., Farmer S.F., Nielsen J.B. (2017). A critical period of corticomuscular and EMG-EMG coherence detection in healthy infants aged 9–25 weeks. J. Physiol..

[15] Clowry G.J. (2007). The dependence of spinal cord development on corticospinal input and its significance in understanding and treating spastic cerebral palsy. Neurosci. Biobehav. Rev..

[16] Hennequin R., Khlif A., Voituret F., Moussallam M. (2020). Spleeter: A fast and efficient music source separation tool with pre-trained models. J. Open Source Softw..

[17] Pons C., Brochard S., Grigoriu A., Newman C.J., Monbaliu E., Mensah-Gourmel J., Gaudin-Drouelle D., Toumi A., Konings M., de la Cruz J. (2023). Digital technologies for motor rehabilitation in children: Protocol for a cross-sectional European survey. BMJ Open.

[18] World Health Organization (2001). International Classification of Functioning, Disability and Health: ICF.

[19] Cegarra B., Cattaneo G., Ribes A., Solana-Sanchez J., Sauri J. (2023). Independent living, emotional well-being, and quality of life in people with disabilities: The mediator role of self-determination and satisfaction with participation. Front. Psychol..

[20] Merchant Z., Troland E., Webber D. (2024). The Hidden Cost of Disability.

[21] Novosel I.B., Ritterband-Rosenbaum A., Zampoukis G., Nielsen J.B., Lorentzen J. (2023). Accurate Monitoring of 24-h Real-World Movement Behavior in People with Cerebral Palsy Is Possible Using Multiple Wearable Sensors and Deep Learning. Sensors.

[22] Mishra S., Laplante-Levesque A., Barbareschi G., Witte L., Abdi S., Spann A., Khasnabis C., Allen M. (2024). Assistive technology needs, access and coverage, and related barriers and facilitators in the WHO European region: A scoping review. Disabil. Rehabil. Assist. Technol..

[23] Doherty C., Baldwin M., Keogh A., Caulfield B., Argent R. (2024). Keeping Pace with Wearables: A Living Umbrella Review of Systematic Reviews Evaluating the Accuracy of Consumer Wearable Technologies in Health Measurement. Sports Med..

