# Supplementary material for: New Method to Motivate Participation in Daily Life/Everyday Life Activities Using Sensor-Based Smart Application Translating Intention into Action (TIA)
Source: Sensors (Basel). 2026 Jan 13;26(2):539. doi: 10.3390/s26020539 (PMC12845928; doi:10.3390/s26020539)
Supplement: Supplementary file 1 [file sensors-26-00539-s001.zip › Supplementary Material System Manual S1.pdf]

User guide for Translating Intension into Action (TIA)

The TIA application (formerly known as MusicMotion) enables the activation of sounds, music, or video through motion sensors, where the motion threshold can be adjusted according to the user's functional needs.

The app exists in two different versions, the latest of which has been further developed to enable multiple users to play music together at the same time.

**Start**<br>Open TIA app	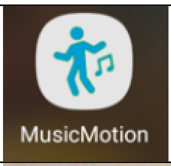
**Connecting sensors to the app**<br>Turn on the sensors (if Movesense) by holding a finger on both metal screws to activate until a red light comes on.<br><br>Hold the sensors close to the screen until four symbols appear in the app.<br><br>The next time you open the app, the sensor will be saved, but must be activated again by holding the sensor against the screen until the symbol changes from transparent to colored.	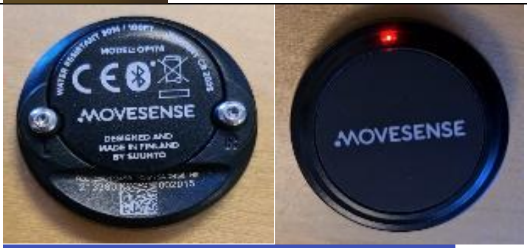<br>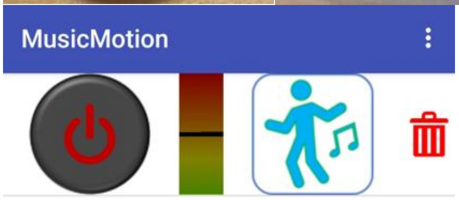<br>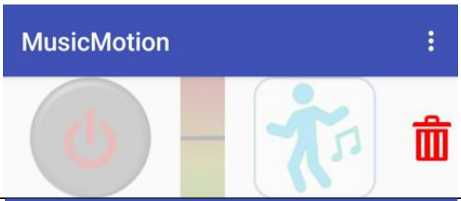
**Sensors**<br>- Red means that the sound/action for the sensor is disabled<br>- Green means that the sound/action for the sensor is enabled	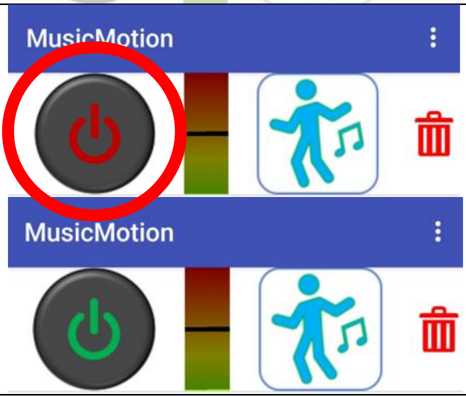

**Calibration column**<br>The color scale indicates sensor movement, and the black line represents the threshold<br><br>To set the threshold to a desired movement, hold a finger on the color scale for 2-5 seconds while performing the desired movement with the sensor, causing the background to turn turquoise during calibration. When you release your finger from the color scale, the app will have calibrated the level of movement based on the information it has received about the movement of the sensor.	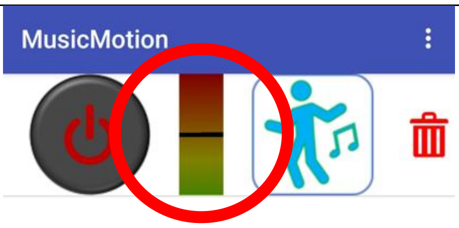
**Selecting an activity**<br>Tap the symbol with the dancing person to select an activity.	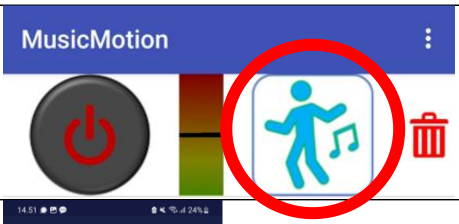
**Select Sound, Music, and Multitrack**<br>The remaining options are Volume, YouTube, Arduino, and ADB, which can be selected if the three options above are not sufficient.	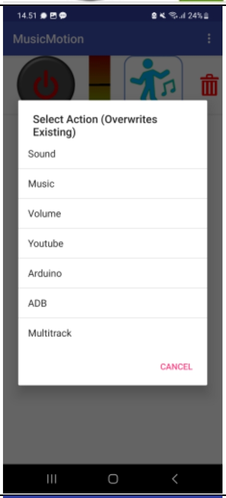
**Sound:**<br>Select an audio file from the list or press ADD to add new MP3 files.<br>If you want to download MP3 files from your device, place the files in the Download folder, as this is often the first folder you access.<br>Once you have pressed an audio file, it is selected and you can press back to return to the main menu.	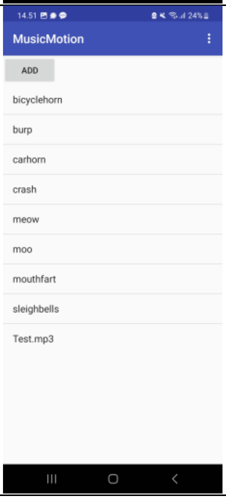

**Music**<br>Select a music file from the list or press ADD to add new audio files from folders on the device.<br>When you press an audio file, the music starts playing, and you can press back to the main menu, where the audio file restarts when you activate the sensor.	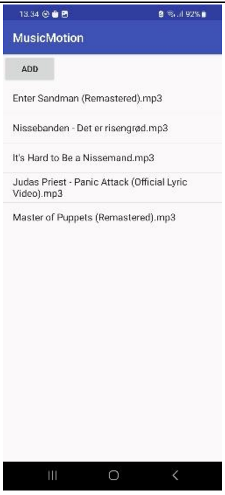<br><br>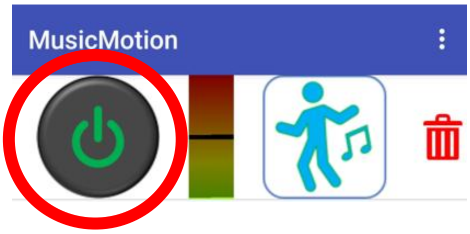
Multitrack:	
**Delete**<br>Trash can for deleting sensors	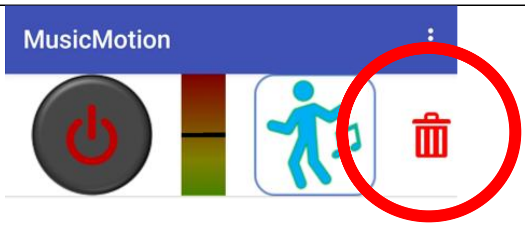
**Advanced settings**<br>If you want to set the threshold more accurately, you can go to advanced settings by:<br>- Pressing the three dots<br>- Pressing Toggle Advanced Settings<br>- Holding your finger on the sensor symbol for 3 seconds.<br>This gives you access to a wide range of different settings options.	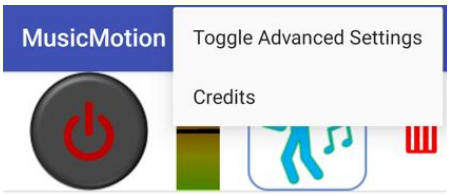<br><br>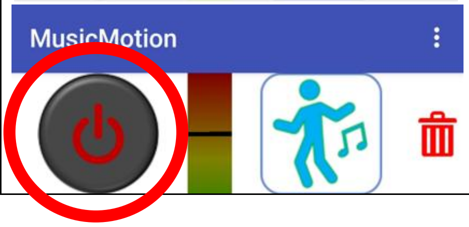

<table>
<tr>
<td>

Setting the threshold

The coordinate system displays a blue line indicating the sensor's movement and a red line indicating the threshold for activating an activity.

</td>
<td>

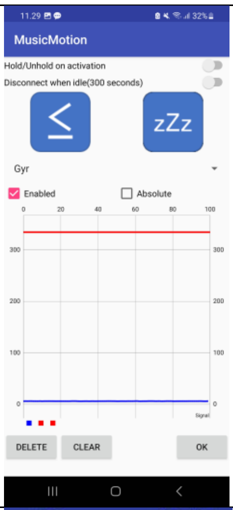

</td>
</tr>
<tr>
<td>

When the sensor moves away from the desired movement, you will see a fluctuation in the blue line.

</td>
<td>

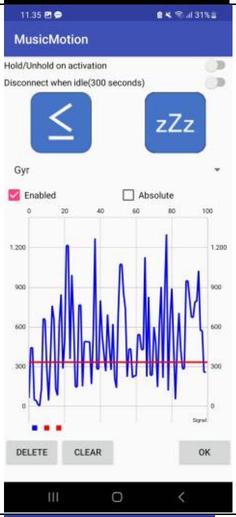

</td>
</tr>
<tr>
<td>

To select the activation threshold, quickly press the screen based on the level of movement you want.

If the threshold is set too low, you will experience frequent activation at lower movement levels, and if the threshold is set too high, you will find that greater movement is required for activation.

Confirm and save your settings by pressing OK to return to the start menu.

</td>
<td>

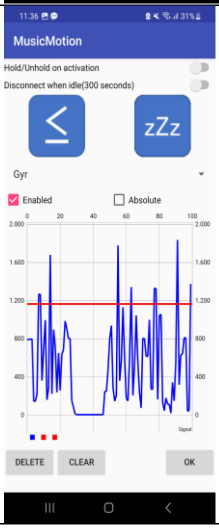

</td>
</tr>
</table>

**Setting the time frame for activation before reactivating an activity.**<br>- By pressing the zZz symbol, you can set how many milliseconds must pass before a given activity can be reactivated.<br>- If the level is set low, you may experience, for example, that a sound is interrupted by the playback of the next sound or by the replay of the same sound.<br>- If the level is set high, you may experience that the sound is not activated when you want it to be, as not enough time has passed since the last activation.<br>- It is therefore important to find a balance in the setting so that the user experiences a connection between the movement they produce and the activation of the sound.<br>- This setting option may be relevant if you:<br>o Experience many large involuntary movements, causing you to reactivate the sound.<br>o You experience latency during the activation of voluntary movements, causing you to reactivate the sound before you feel a connection between the desired movement and the activation of the sound.	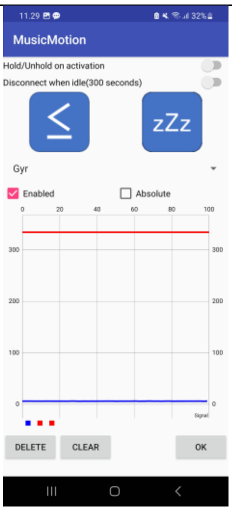
**Additional settings**<br>Enable activation<br>- (Added under motion selection)<br>Defining thresholds<br>- By pressing ≤, you can define specific thresholds instead of pressing the coordinate system.<br>- Specific thresholds can also be read here.<br>Hold/unhold on activation<br>- When this option is activated, you can define whether the sound for “Hold off” should be activated each time the movement is performed, or whether “Hold on” should be activated in a loop with one movement of the sensor and terminated with the next movement of the sensor.<br>Disconnect when idle 300 seconds<br>- The sensor is deactivated after 5 minutes. - The sensor is deactivated after 5 minutes.	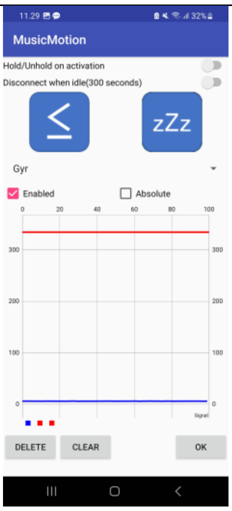
